# Long-Term Results After Salter Innominate Osteotomy for the Treatment of Developmental Dysplasia of the Hip—Only 8% Rate of Total Hip Arthroplasty at a Median Follow-Up of 22 Years †

**DOI:** 10.3390/children11121525

**Published:** 2024-12-16

**Authors:** Renée Anne van Stralen, Ena Colo, Erich Rutz, Berend Willem Schreurs, Allard Jan Frederik Hosman

**Affiliations:** 1 Department of Orthopedics, Radboud University Medical Center, 6525 GA Nijmegen, The Netherlands; ena.colo@radboudumc.nl (E.C.); wim.schreurs@radboudumc.nl (B.W.S.); allard.hosman@radboudumc.nl (A.J.F.H.); 2 Department of Orthopedics and Sports Medicine, Erasmus Medical Center, 3015 GD Rotterdam, The Netherlands; 3 Department of Paediatric Orthopaedic Surgery, The Royal Children’s Hospital, Melbourne 3052, Australia; erich.rutz@rch.org.au; 4 Murdoch Children’s Research Institute (MCRI), Melbourne 3052, Australia; 5 Department of Paediatrics, Bob Dickens Chair, Paediatric Orthopaedic Surgery, The University of Melbourne, Melbourne 3010, Australia; 6 Medical Faculty, University of Basel, 4001 Basel, Switzerland; 7 School of Health and Biomedical Sciences, Royal Melbourne Institute of Technology (RMIT University), Melbourne 3000, Australia

**Keywords:** hip dysplasia, pelvic osteotomy, total hip arthroplasty

## Abstract

Background/Objectives: The redirection or reshaping of the acetabulum might be warranted to attain a concentric and stable hip in children with developmental dysplasia of the hip (DDH). The aim of this study is to assess the late clinical and radiological results, and to determine the number of patients requiring secondary surgery or a total hip arthroplasty at a long-term follow-up. Methods: Our institution performed 99 Salter osteotomies on 76 patients without underlying neuromuscular conditions over a 21-year period, from 1981 to 2002. These procedures were carried out by three different surgeons. Patients underwent a comprehensive evaluation at the review visit, including a physical examination, clinical assessments using the Harris hip score (HHS), Oxford score (OHS) and Visual Analogue Scale (VAS) pain score, as well as pelvic radiographs. Results: At a median follow-up of 22 years, total hip arthroplasty was performed in 6 out of 77 hips (8%). Patients who underwent a Salter osteotomy with an open reduction had a higher rate of avascular necrosis (AVN) of the femoral head compared to those who only underwent a Salter pelvic osteotomy (*p* < 0.001). There were statistically significant differences in the group with and without AVN in terms of HHS (*p* = 0.006, 95%CI 0.003 to 0.006), OHS (*p* = 0.016, 95%CI 0.012 to 0.017), a modified OHS (*p* = 0.018, 95% CI 0.012 to 0.016), a VAS score in activity (*p* = 0.046, 95%CI 0.042 to 0.050) and VAS score satisfaction (*p* = 0.005, 95%CI 0.003 to 0.006). Conclusions: The rate of THA was 8% at a mean of follow-up of 22 years. Secondary results suggest that AVN occurs more frequently when a Salter osteotomy is combined with an open reduction. The occurrence of AVN is associated with significantly lower clinical outcome scores and patient satisfaction, as well as significantly higher pain scores. In the absence of AVN, good clinical results can be expected at long-term follow-up.

## 1. Introduction

**Figure 1:**
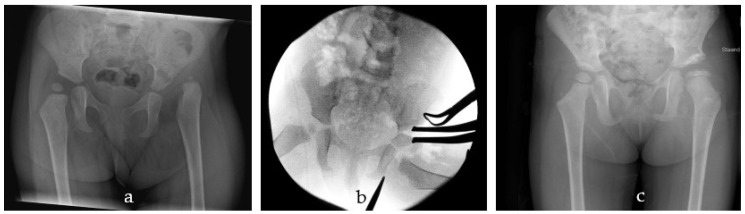
Radiographs of a Salter pelvic osteotomy. (**a**) Preoperative radiograph showing a late diagnosis of a dislocated hip in a girl aged 1 year and 9 months. (**b**) Intra-operative radiograph of pelvic osteotomy and open reduction at age 2 years. (**c**) Postoperative results after 2 years, showing postoperative avascular necrosis of the femoral head.

Developmental dysplasia of the hip (DDH) can be defined as the insufficient acetabular coverage of the femoral head. It can range from mild dysplasia to instability of the hip. The goal of primary treatment of DDH is to obtain a concentric hip reduction and to facilitate the remodelling of the acetabulum. Primary treatment consists of conservative treatment with an abduction splint and, if that fails, a closed reduction [1,2,3]. If conservative treatment fails, surgical management can be considered. One of the most commonly performed procedures used to stabilise the hip and correct the acetabular coverage is the Salter pelvic osteotomy [4]. The rationale for the Salter innominate osteotomy is the centring and stabilisation of the femoral head by redirecting the acetabulum (Figure 1) [4,5,6]. 

Several reports describe mid- to long term follow-up after a Salter osteotomy [7,8,9,10,11,12,13,14,15]. Furthermore, a recent systematic review included pooled data for 1372 hips after a Salter pelvic osteotomy in 36 studies [16]. Comparing the mid- and long-term results of several types of pelvic osteotomy, this systematic review reports favourable outcomes for the Salter pelvic osteotomy both clinically (in 86% of hips) and radiologically (in 84% of hips) [16].

Salter pelvic osteotomy has been performed by paediatric orthopaedic surgeons at our academic institute (RadboudUMC, Nijmegen, the Netherlands) since 1980. In this study, we examined the need for secondary reconstructive surgery and the need for total hip arthroplasty in 99 Salter innominate osteotomies performed in 76 patients without underlying neuromuscular pathology at our institution. The purpose was to assess the late clinical and radiological results, and to assess the number of patients requiring secondary surgery or total hip arthroplasty (THA).

## 2. Materials and Methods

The surgical technique used for the Salter pelvic osteotomy at our institution was performed as described by Salter in the original publication [4,5]. Following the standard protocol, patients were fitted with a spica cast postoperatively, with the hip immobilised for 6 weeks. When an open reduction was performed simultaneously, the immobilisation period was extended to 12 weeks. The osteotomies were performed by three surgeons and the surgical technique was the same over the study period. 

### 2.1. Patient Selection

Ethical board approval was obtained prior to this study (NL51879.091.14). Surgical records were reviewed and all patients that underwent a Salter pelvic osteotomy were selected. All patients who were older than 18 years at the time of start of the review study and who had no underlying (neuromuscular) condition were selected. Between January 1981 and December 2002, 99 Salter pelvic osteotomies had been performed in 76 patients. In most cases, DDH was established by radiography. These patients were included as eligible for follow-up. Information on surgical and postoperative management, as well as postoperative complications, were taken from the medical records. Postoperative complications were classified using the Clavien–Dindo system [17]. 

Patients were approached for clinical follow-up by telephone or via a letter and subsequently seen in clinic. They had a clinical examination and responded to several questionnaires, such as the Harris hip score (HHS) [18], the Merle d’Aubigne score [19] and the Oxford hip score [20]. Furthermore, they filled out a Visual Analogue Scale score with regard to pain at rest, pain during activities and a VAS satisfaction score, as validated by Brokelman et al. [21].

Radiographs at the time of follow-up were analysed to determine the centre edge angle of Wiberg (CE angle) [22], the caput–collum–diaphyseal angle (CCD angle) [23], the presence of avascular necrosis (AVN) of the femoral head or proximal femoral growth disturbance (PFGD) [24], the Severin classification [25], the Kellgren and Lawrence classification [26] and the Crowe classification [27]. 

Functional results were evaluated by one of the authors (E.C.). Radiographic measurements were carried out by two authors (E.C. + R.v.S.) and scored on an agreement basis. Any disagreements were solved by involving the third author and performing radiographic measurements together (WS). 

### 2.2. Statistical Analysis

All data analysis was performed using IBM^®^ SPSS software, version 28.0 (SPSS Inc., Chicago, IL, USA). Significance was defined as *p* < 0.05. 

The distribution of data were assessed using the Kolmogorov–Smirnov test and the Shapiro–Wilk test. Continuous outcome parameters are presented as median and range or interquartile range (IQR) depending on data distribution and dichotomous measures as counts and percentages. Correlation coefficients were performed using Spearman’s correlation analysis. To compare continuous outcome parameters per group (centre edge angle at follow-up per age group at surgery, clinical outcome scores in the group with AVN vs. without AVN, clinical outcome scores in the group with or without an open reduction), a non-parametric Mann–Whitney U test was used based on data distribution. 

To compare the number of cases with AVN in the group with or without an open reduction at the time of surgery, a cross table with Fisher’s exact test was used, as there were cells with less than five cases. 

Survival curves were performed using a Kaplan–Meier curve.

Sensitivity analyses were performed to assess the baseline characteristics (median age at surgery, surgeon, year of surgery, duration of available follow-up, salter alone vs. combination with open reductions) for the included and excluded patients. Frequencies were compared using a chi-square test and medians were compared using a nonparametric Mann–Whitney U test. The outcomes to these analyses are included in the Supplementary Materials.

## 3. Results

### 3.1. Patient Inclusion

**Figure 2:**
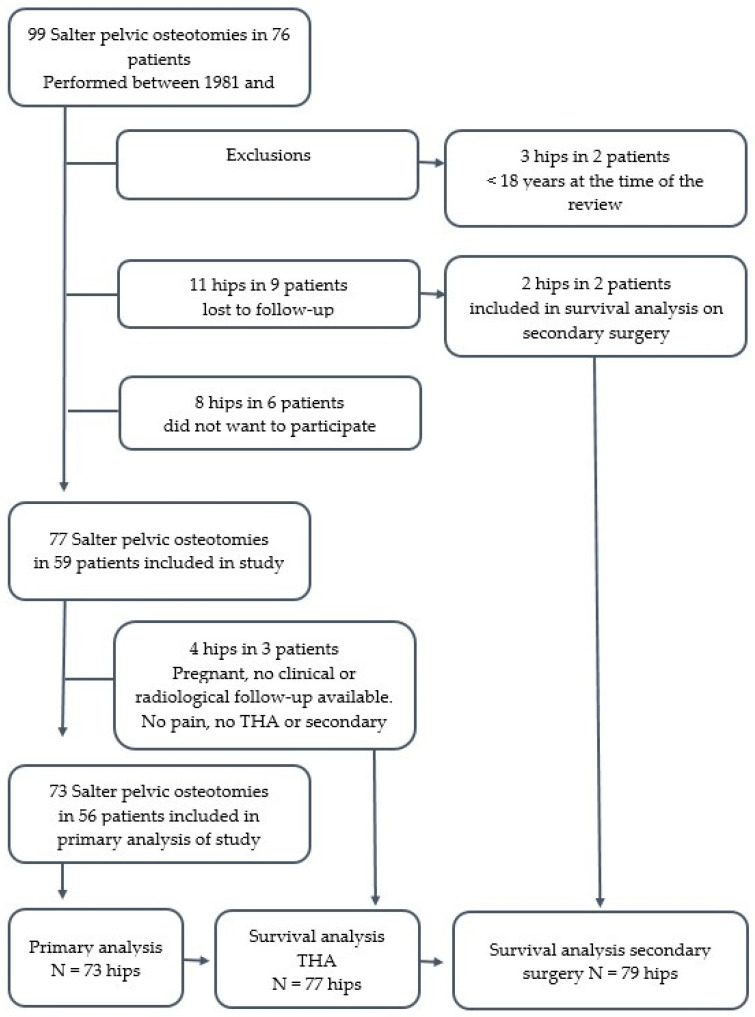
Flowchart of patient inclusion.

Two patients (three hips) were excluded as they had not reached the age of 18 years at the time of the review. A total of 15 patients (19 hips) were not available for review; of these, 9 patients (11 hips) were lost to follow-up and 6 patients (8 hips) did not want to participate in the study. In addition, three patients (four hips) were pregnant at the time of the follow-up. These three patients reported by phone to have experienced no pain, nor had they undergone a reoperation for THA; however, no scores and radiographs were obtained. Their results were excluded from functional analysis, but these four hips were included in the survival analysis. For two of the hips that were lost to follow-up, there was information regarding secondary surgery, so they were included in the survival curve that looked at revision surgery (Figure 2).

Thus, the studied cohort is based on 59 patients (77 hips). Of these 59 patients, in 51 patients (64 hips), the clinical scores were available. Radiographs were available in 52 patients (65 hips).

In 45 patients (56 hips), both clinical and radiological follow-up was available. For 73 hips in 57 patients there was either a radiological follow-up or a clinical follow-up available. These patients were included in the main analyses of this study. A total of 77 hips were included in the survival analysis, which looked at THA as an endpoint, and 79 hips were included in the survival curve, which looked at secondary surgery as an endpoint.

### 3.2. Baseline Characteristics

There were 10 male patients (13 hips) and 47 female patients (60 hips). The median age at time of surgery was 3.4 years (IQR 2.6–4.3 years). In 19 hips, there was a positive family history for developmental dysplasia of the hip (DDH). The baseline characteristics are depicted in Table 1. 

The median duration of surgery was 1 h and 40 min in the group that underwent a Salter osteotomy alone (range of 45 min to 3 h and 40 min), and the median duration of surgery was 2 h and 30 min in the group that underwent an open reduction with a Salter pelvic osteotomy (range of 1 h and 30 min to 4 h and 40 min). 

### 3.3. Preoperative Treatment

With regard to treatment prior to Salter pelvic osteotomy, 30 children had undergone a previous open reduction and 16 children had undergone a previous closed reduction. Eight patients had different types of bony surgery prior to their Salter osteotomy; three patients had a femoral osteotomy, three patients had a previous Salter osteotomy at a different institution and two patients had undergone a different type of pelvic osteotomy.

In 42 cases, the patient had undergone treatment with an abduction splint. In 41 cases, the patients received overhead traction prior to their pelvic osteotomy. In 15 cases, the operation was performed because of a dislocated hip, and in 1 case, because of subluxation. The median acetabular index for the overall population was 30° (IQR 26°–34°). Surgical indication at the clinic appointment was a dislocated hip in 16 cases and residual dysplasia in 57 hips. In 29 cases, an open reduction was performed concurrently with the Salter osteotomy due to persistent instability or intraoperative factors. These intraoperative factors included the persistent lateralization or instability of the femoral head following pelvic osteotomy. In this group, the median preoperative acetabular index was 35° (IQR 25°–39°). In the group of patients that underwent a Salter osteotomy without an additional open reduction (n = 44), the median preoperative acetabular index was 29° (IQR 26°–32°).

In eight cases, Salter osteotomy was performed on patients under the age of 1.5 years (range of 0.8–1.4 years). In all of those cases, an open reduction was performed at the same time. The median acetabular index in this group was 38° (IQR 30°–40°).

Only five patients had not undergone any form of prior treatment. There was no significant difference in age at surgery between the group of patients that underwent treatment prior to Salter pelvic osteotomy or in those that underwent Salter osteotomy as the first treatment for their dysplasia (*p* = 0.84, 95% CI 0.83 to 0.84). 

### 3.4. Postoperative Complications

In 22 hips, postoperative complications occurred in the direct postoperative phase (Table 2). In three hips, avascular necrosis of the femoral head was acknowledged within the first 3 months post operation.

### 3.5. Radiological Follow-Up

At the final radiological follow-up, the median CE angle was 33° (IQR 27°–42°). The median caput–collum–diaphyseal angle was 136° (IQR 132°–140°). The radiological classifications are depicted in Table 3.

There was no significant difference in CE angle at final follow-up when looking at the children that underwent surgery aged 0–4 and 4 years and older (both groups had a median of 33 degrees, *p* = 0.5, 95% CI 0.538 to 0.557). 

In 13 hips, AVN was present at final follow-up. Eleven of these hips had undergone an open reduction, so the population of patients that had an open reduction during the Salter osteotomy procedure exhibited a significantly higher rate of AVN in (*p* < 0.001).

### 3.6. Clinical Follow-Up

The median follow-up was 23 years (range 13–34 years). The median BMI at final follow-up was 23 kg/m^2^ (IQR 21–25 kg/m^2^). Clinical outcomes measures at follow-up are depicted in Table 4a,b. 

At the final follow-up, 9 hips (12%) had a HHS of < 70, and 55 hips had a good clinical outcome with a HHS of > 70 (75%) (9 missing hips, 12%). There were no significant differences in outcome parameters between children that underwent a Salter osteotomy alone and those that underwent a Salter osteotomy combined with an open reduction (Table 4a). 

There were statistically significant differences in clinical outcome between the population showing signs of AVN and those without AVN. In the group with AVN, the Harris hip score was significantly lower, the Oxford hip score was significantly higher, the Modified Oxford hip score was significantly lower, the VAS score during activity was significantly higher and the VAS satisfaction score was significantly lower (Table 4b). 

There were no significant differences in clinical outcome measures in terms of children that underwent surgery aged 0–4 or aged 4 years and older. 

### 3.7. Additional Reconstructive Surgery

**Figure 3:**
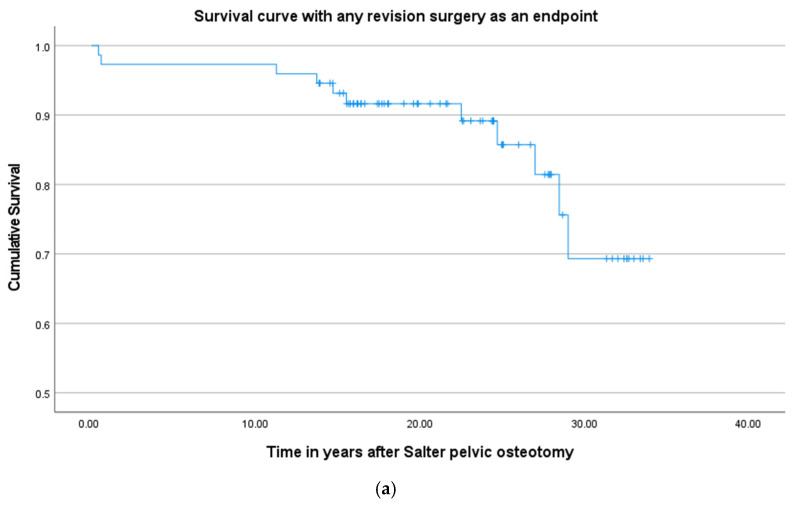

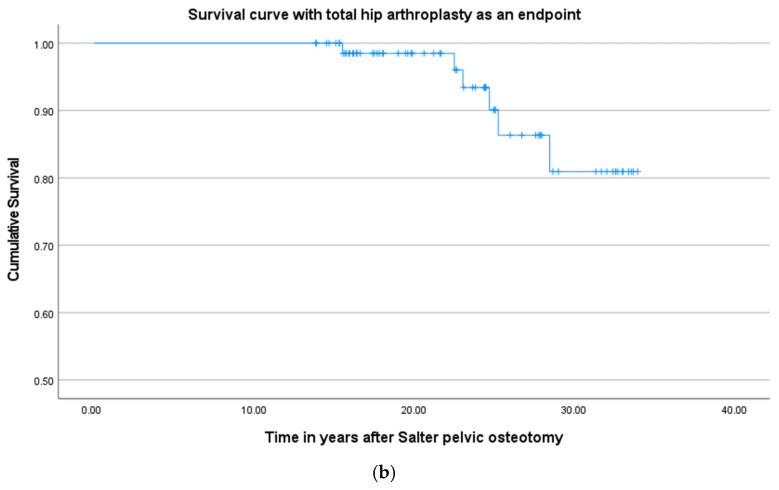
Survival curves: (**a**) survival curve with any additional surgery as an endpoint; (**b**) survival curve with total hip arthroplasty as an endpoint.

Of the 79 hips included in the survival analysis looking at secondary surgery, 10 hips underwent secondary surgery before the final follow-up. Three hips underwent an additional Varus Derotation Osteotomy (VDRO). Seven hips underwent an additional pelvic osteotomy for residual dysplasia; of these, three hips underwent a re-do Salter pelvic osteotomy, one hip underwent a Pemberton osteotomy, one hip underwent a triple pelvic osteotomy and two hips underwent a Serafimov procedure (Figure 3a) [28].

### 3.8. Total Hip Arthroplasty 

Of the 59 patients (77 hips) included in the survival analysis looking at THA, 6 hips underwent total hip arthroplasty before the final follow-up. The median duration between Salter osteotomy and total hip arthroplasty was 24 years (IQR 21–26 years). The median age at total hip arthroplasty was 28 years (IQR 24–30 years). 

A survival curve is depicted in Figure 3b.

## 4. Discussion

This study is one of the few that describes a long-term clinical and radiographic follow-up of 99 hips in 75 healthy children without underlying neuromuscular pathology.

At a median follow up of more than 22 years after the surgery, most patients had a satisfying outcome and 58 patients (71 hips) required no further surgery to improve acetabular coverage. This is comparable to other studies with a similar follow-up [7,8,9,10,11,12,13,14,15,16].

Sensitivity analyses looking at the baseline characteristics included in the Supplementary Materials show that there were no significant differences in the percentage of patients that underwent an additional open reduction in the included group and the excluded group. This implies that the missing data are random and that the results can be interpreted without further bias.

The technical complications observed in our cohort were of a similar order of magnitude compared to those reported in the existing literature. Salter and Dubos reported that 5.6% of the hips in their series redislocated and 14.3% resubluxed [29]. Other investigators have also reported redislocation or resubluxation rates of 2% to 14% [7,30,31,32]. The present cohort had one case of redislocation (1%) requiring additional surgery. 

The prevalence of AVN after a Salter pelvic osteotomy has been reported to range from 2.2% to 10%. [7,12,33,34,35] After an open reduction that risk can increase up to 13% [32,36] with late radiographic signs of AVN reaching up to 34.6% [10]. In our cohort, the prevalence of AVN at review was significantly higher in the population that underwent an open reduction combined with a Salter osteotomy (43%) compared to the population that underwent only a Salter osteotomy (5%) (*p* < 0.001), but this is very similar to the prevalence of 44% PFGD found by Kiani et al. [37]. The prevalence of AVN in the population that underwent a Salter osteotomy is comparable to the results in other studies, whereas the percentage of AVN described in the population that underwent an additional open reduction is relatively high compared to the results found in the literature. 

The clinical outcome appears to be significantly worse in patients with AVN with regard to Harris hip score, Oxford hip score, modified Oxford hip score, VAS score in activity and VAS satisfaction score. These differences are relevant because the differences in patient outcome appear to be bigger than the influences on outcome as a result of age at the time of surgery or a patient having undergone an additional open reduction. This study therefore confirms Salters’ theory that AVN is a complication of open reduction rather than of osteotomy [28]; this was also confirmed by Böhm et al. [7]. In addition, this suggests that the clinical outcomes in terms of functional scores and satisfaction scores are significantly lower and pain scores are significantly higher when AVN occurs. 

In this study, 10 of the 79 hips included in the survival analysis underwent secondary reconstructive surgery before the final follow-up. Total hip arthroplasty was performed on 6 out of 78 hips (8%). Malvitz et al. described a slightly higher percentage of hip replacements (11.8%) at a follow-up of 30 years, but their cohort consisted of only congenital hip dislocations [38]. Thomas et al. described a much higher rate of 31% THA in 77 hips at a minimum follow-up of 40 years and mentioned an additional 17% of patients with definite osteoarthritis [39]. In a different paper, Thomas noted that at 33 years, the rate of hip arthroplasty was relatively low at 3.8% [13]. However, by 45 years, 46% of patients had undergone THA [13]. This population, however, consisted of only late-presenting congenital hip dislocations and had a longer follow-up than the present study, which could explain the higher rate of hip replacements. Bohm et al. reported on 73 Salter pelvic osteotomies at a mean follow-up of 30.9 years and identified a slightly lower (6.8%) rate of THA at final follow-up [7]. 

The present study is limited by its retrospective design and the number of patients lost to follow-up. However, because of the long-term follow-up available in up to 80% of the included hips, we do feel that the results of this study are valuable. Furthermore, sensitivity analyses revealed no bias in patients lost to follow-up. Another limitation of our study is that due to its retrospective design, the lack of standardisation in outcome evaluation methods over time could lead to selection bias and the presence of confounding variables, which may affect the generalizability and interpretation of the results. A final limitation of this study is the relatively small sample size of 59 patients. This could limit the statistical power of our analysis and may reduce our ability to detect smaller differences in outcomes. Future studies with larger cohorts are needed to further validate our findings and explore more nuanced differences

## 5. Conclusions 

In conclusion, our study demonstrates the long-term outcomes of Salter pelvic osteotomies performed in children with DDH, showing low rates of total hip arthroplasty (8%) at final follow-up, which corroborates findings from previous studies [7,8,9,10,11,12,13,14,15,16,32,33,34,35]. Furthermore, the secondary outcomes of this paper suggest that AVN occurs more frequently after Salter pelvic osteotomy combined with an open reduction than after Salter pelvic osteotomy alone. When AVN occurs, patient-reported outcome scores and patient satisfaction are significantly lower and pain scores are significantly higher. When a concentric, well-reduced hip can be achieved with good acetabular morphology, good clinical results can be expected at long-term follow-up. 

Additional prospective research is required to further investigate the long-term outcomes of Salter pelvic osteotomies used for the management of developmental dysplasia of the hip. 

## Figures and Tables

**Table 1:** Baseline characteristics.

	Number of Hips
Male/Female	13:60
Left/Right	38:35
Age; Median (range)	3.4 years (IQR 2.6–4.3 years)
Prior treatment	
Traction	41 (56%)
Abduction splint	42 (58%)
Closed reduction +/− adductor/psoas tenotomy	16 (22%)
Open reduction +/− adductor/psoas tenotomy	30 (41%)
Salter at different institution	3 (4%)
Femoral osteotomy	3 (4%)
Different type of pelvic osteotomy	2 (3%)
Median preoperative acetabular index	30° (IQR 26°–34°)
Instability/residual dysplasia	16:57
Concomitant procedures	52 (71%)
Open reduction	29 (40%)
Psoas tenotomy	48 (66%)
Adductor tenotomy	4 (6%)
VDRO	9 (12%)
Fixation method used	
K-wire	57 (78%)
Absorbable pin	16 (22%)
Median duration of surgery in Salter group	1:40 (range 0:45–3:40)
Median duration of surgery in Salter + open reduction group	2:30 (range 1:30–4:40)
Median duration of admission	9 (IQR 6–13 days)
Median duration of available follow-up	22 years (range 13–34)
Median BMI at follow-up	23 kg/m^2^ (range 16–39)

**Table 2:** Postoperative complications.

Clavien Dindo Classification [17]	Type of Complication	Number of Hips
Grade I	Recurrent instability without requiring additional surgery;	1 (1%)
	Avascular necrosis of the femoral head.	3 (4%)
Grade II	Fever without a cause, requiring treatment;	9 (12%)
	Superficial infection;	3 (4%)
	Delirium.	1 (1%)
Grade III		
Grade IIIa	Fracture; ^2^	4 (5%)
Grade IIIb	Deep infection. ^1^	1 (1%)
Grade IV		
Grade IVa		
Grade IVb		
Grade V		

^1^ Treated by surgical debridement and antibiotics. ^2^ There were four distal femoral fractures after the removal of the spica casts. Three cases were treated in an above knee cast, one was managed by traction.

**Table 3:** Summary of radiological outcome parameters.

	Number of Hips	Percentage
Severin classification [25]		
1	42	58%
2	11	15%
3	9	12%
4	2	3%
5	1	1%
Missing	8	11%
AVN ^1^ or PFGD ^2^ present		
No	53	73%
Yes	13	18%
Missing	7	10%
Kellgren–Lawrence classification [26]		
0	32	44%
1	21	29%
2	3	4%
3	7	10%
4	2	3%
Missing	8	11%

^1^ AVN—avascular necrosis; ^2^ PFGD—proximal femoral growth disturbance.

**Table 4:** Clinical outcome scores. (**a**) Median outcome (and range) of clinical evaluation scores between the total population, the group that just underwent a Salter pelvic osteotomy and the group that underwent a Salter pelvic osteotomy combined with an open reduction. (**b**) Median outcome of clinical evaluation scores between the group that showed signs of AVN at final follow-up and the group that did not.

**(a)**						
	**Total Popu-lation**	**Salter Osteotomy**		**Salter Osteotomy + Open Reduction**	**Significance**	**95% Confidence Interval**
Harris hip score [18] (range)	100 (50–100)	100 (50–100)		98 (56–100)	*p* = 0.5	0.38 to 0.39
Oxford hip score [20] (range)	13 (12–49)	12 (12–49)		13 (12–30)	*p* = 0.8	0.63 to 0.65
Modified Oxford hip score (range)	47 (11–48)	48 (11–48)		47 (30–48)	*p* = 0.9	0.88 to 0.89
VAS ^1^ score in rest in mm (range)	0 (0–60)	0 (0–60)		0 (0–45)	*p* = 0.3	0.45 to 0.47
VAS score in activity in mm (range)	0 (0–85)	0 (0–85)		3 (0–80)	*p* = 0.7	0.63 to 0.65
VAS score satisfaction in mm [21] (range)	80 (10–100)	90 (20–100)		80 (10–100)	*p* = 0.5	0.36 to 0.38
**(b)**						
	**AVN ^2^ Present** **N = 14 (19%)**		**No AVN Present** **N = 53 (73%)**		**Significance**	**95% Confidence Interval**
Harris hip score [18] (range)	93 (51–100)		100 (64–100)		*p* = 0.006	0.003 to 0.006
Oxford hip score [20] (range)	16 (12–35)		12 (12–49)		*p* = 0.016	0.012 to 0.017
Modified Oxford hip score (range)	45 (25–48)		48 (11–48)		*p* = 0.018	0.012 to 0.016
VAS score in rest in mm (range)	0 (0–45)		0 (0–60)		*p* = 0.081	0.045 to 0.053
VAS score in activity in mm (range)	20 (0–85)		0 (0–80)		*p* = 0.046	0.042 to 0.050
VAS score satisfaction in mm [21] (range)	70 (10–100)		90 (20–100)		*p* = 0.005	0.003 to 0.006

^1^ VAS score—Visual Analogue Scale, measured in centimetres; ^2^ AVN—avascular necrosis.

## Data Availability

The data presented in this study are available on request from the corresponding author. The data are not publicly available due to security and confidentiality concerns.

## Supplementary Materials

The following supporting information can be downloaded at: https://www.mdpi.com/article/10.3390/children11121525/s1. Supplement S1: Sensitivity analyses.
