# Supplementary material for: Long-Term Results After Salter Innominate Osteotomy for the Treatment of Developmental Dysplasia of the Hip—Only 8% Rate of Total Hip Arthroplasty at a Median Follow-Up of 22 Years
Source: Children (Basel). 2024 Dec 16;11(12):1525. doi: 10.3390/children11121525 (PMC11727591; doi:10.3390/children11121525)
Supplement: Supplementary file 1 [file children-11-01525-s001.zip › children-3347842-Supplementary S1.pdf]

Supplement – Sensitivity analyses

Age at surgery

Cases included in clinical follow-up

	Excluded from clinical follow-up<br>N = 36	Included in clinical follow-up<br>N = 64
Median age at time of surgery	3.8	3.4

Nonparametric T test p = 0.182

Cases included in radiological follow-up

	Excluded from radiological follow-up<br>N = 33	Included in radiological follow-up<br>N = 66
Median age at time of surgery	4.0	3.3

Nonparametric T test p = 0.009

Age at surgery – divided into age categories

Cases included in clinical follow-up

	Excluded from clinical follow-up<br><br>N = Number of patients<br>% Percentage of excluded patients	Included in clinical follow-up<br><br>N = Number of patients<br>% Percentage of excluded patients	
Age at surgery 0-4 years	N = 20 (57.1%)	N = 39 (60.9%)	N = 59
Age at surgery > 4 years	N = 15 (42.9%)	N = 25 (39.1%)	N = 40
	N = 35	N = 64	

Chi square test: P = 0.713

Cases included in radiological follow-up

	Excluded from radiological follow-up<br><br>N = Number of patients<br>% Percentage of excluded patients	Included in radiological follow-up<br><br>N = Number of patients<br>% Percentage of excluded patients	
Age at surgery 0-4 years	N = 14 (42.4%)	N = 45 (68.2%)	N = 59

Age at surgery > 4 years	N = 19 (57.6%)	N = 21 (31.8%)	N = 40
	N = 33	N = 66	N =

Chi square test: P = 0.014

Year of surgery

Cases included in clinical follow-up

Number of cases excluded from clinical follow-up: N = 35
Number of cases included in clinical follow-up: N = 64

Chi square test: P = 0.525

Cases included in radiological follow-up

Number of cases excluded from radiological follow-up: N = 33
Number of cases included in radiological follow-up: N = 66

Chi square test: P = 0.329

Open reduction performed

Cases included in clinical follow-up

	Excluded from clinical follow-up<br><br>N = Number of patients<br>% Percentage of excluded patients	Included in clinical follow-up<br><br>N = Number of patients<br>% Percentage of excluded patients	
No open reduction performed	N = 27 (77.1%)	N = 37 (57.8%)	N = 64
Open reduction performed in combination with Salter	N = 8 (22.9%)	N = 27 (42.1%)	N = 35
	N = 35	N = 64	N = 99

Chi square test p = 0.054

Cases included in radiological follow-up

	Excluded from radiological follow-up<br><br>N = Number of patients	Included in radiological follow-up<br><br>N = Number of patients	

	% Percentage of excluded patients	% Percentage of excluded patients	
No open reduction performed	N = 24 (72.7%)	N = 40 (60.6%)	N = 64
Open reduction performed in combination with Salter	N = 9 (27.3%)	N = 26 (39.4%)	N = 35
	N = 33	N = 66	N = 99

Chi square test p = 0.234

Surgeon

<u>Cases included in clinical follow-up</u>

	Excluded from clinical follow-up<br><br>N = Number of patients<br>% Percentage of excluded patients	Included in clinical follow-up<br><br>N = Number of patients<br>% Percentage of excluded patients	
Surgeon 1	N = 31 (88.6%)	N = 51 (79.7%)	N = 82
Surgeon 2	N = 2 (5.7%)	N = 12 (18.8%)	N = 14
Surgeon 3	N = 2 (5.7%)	N = 1 (1.6%)	N = 3
	N = 35	N = 64	

Chi square test p = 0.121

<u>Cases included in radiological follow-up</u>

	Excluded from radiological follow-up<br><br>N = Number of patients<br>% Percentage of excluded patients	Included in radiological follow-up<br><br>N = Number of patients<br>% Percentage of excluded patients	
Surgeon 1	N = 28 (84.8%)	N = 54 (81.8%)	N = 82
Surgeon 2	N = 4 (12.1%)	N = 10 (15.2%)	N = 14
Surgeon 3	N = 1 (3.0%)	N = 2 (3.0%)	N = 3
	N = 33	N = 66	

Chi square test p = 0.920
